# Monthly Summary

**Published:** 1882-11

**Authors:** 


### MONTHLY SUMMARY.

---

*Zinc vs. Babbitt Metal.*—Thirty years ago a partner of mine, Dr. D. H. Goodno, after experimenting with all metals ever used for dental dies, and finding nothing satisfactory, at last thought that "Babbitt metal," then but little known, might answer the purpose. He tried it, and found, to his great relief, that it was just what he needed. We adopted it at once, and after having used it exclusively all these years, can say it is a *perfect thing* for the purpose. And why should it not be? It has all the requirements needed, viz.: *non-shrinking*, *hardness*, *toughness*, *smoothness*, and melting a ta low temperature. Now, while *zinc* is *hard*, its shrinkage is a serious objection. *Type-metal* does not shrink, but is too brittle.

But it is necessary that the Babbitt metal should be made from a correct formula. Much that is sold, while it answers the purpose for which it is generally used, viz.: bearings for machinery, is made in such a manner that it is too soft for dies. Metal that costs less than forty cents per pound will not answer, for it can't be made and sold for less. To ensure a good article, make it yourself, as follows:

Copper, 1 part;
Antimony, 2 parts;
Tin, 8 parts.

Melt in a crucible, *in the order named*, turning off as soon as the tin is dropped in, and remelt. The S. S. White Co. is now making the above formula. For counter die, use seven parts lead and one part tin, and don't turn too hot; coat the die with whiting. For convenience, moisten the sand with sweet-oil, as it is then always ready for use, and there will be no danger of your cast being spoiled from excess of moisture.

I seldom make the second die, even in sharp, irregular, lower cases. The plate will *always* fit the plaster model, and if that is correct, will, of course, fit the mouth. It is time that zinc was banished from dental laboratories, books of instruction and colleges. There is no more need of it than of the "fifth wheel to a coach." On the other hand, it is simply a nuisance, as any one will say after following the above directions half a dozen times.—*L. P. Haskell, (Chicago, Ill). in New England Journal of Dentistry.*

---

*Acute Conjunctivitis Cause by the Electric Light.*—Dr. W. C Rockliffe records, in the *Lancet,* the case of a man who was engaged in adjusting the carbon points of a lamp of 3000 candle power, without wearing the colored glases commonly used to protect the eyes. As an almost daily occurrence the brilliancy of the spark causes more or less paralysis of the retina, or, to quote his own words, "he rarely is able to perceive the people walking on the footpath when descending the ladder from adjusting." Although this affect soon passes off, on this particular occasion, as he regained his power of vision (in about fifteen minutes) it was followed by rapidly increasing lachrymation, photophobia, pain and swelling of the lids, the whole symptoms being developed in thirty minutes. Having suffered from many slight attacks of a similar nature. he applied cold water, which previosuly had relieved him; but the pain and swelling increasing, I saw him the following day, apparently having suffered intense agony during the night. The lids of both eyes were very hot, red, swollen, and brawny, and level with the superciliary ridge, the swelling extending some little distance upward over the forehead. The pain was most acute in and around the eye. On raising the lids (which was a very diffcult operation the photophobia being so exceedingly intense) a considerable amount of lachrymal fluid gushed out. The conjunctival vessels were exceedingly large, and the eyeball a brilliant scarlet; cornea clear. All these symptoms yielded to a brisk purge and lead lotion in forty-eight hours. His fellow workmen was similarly affected, but to a less extent.

---

*Nitrous Oxide.*—Further experience has not changed the relative position or much enlarged the sphere of action of nitrous oxide. That it is the safest of all anæsthetics has been established beyond a question. In one institution where such administration is subject of record, this gas has been given over 100,000 times, and not only without a death, but without causing in a single instance symptoms sufficiently serious to necessitate transporting the patient home in a carriage. In the city of Philadelphia alone, it has been given over 133,000 times without a death, and without any injurious result. Death cannot be justly attributed to it in more than four cases since its introduction.—*F. C. Reeve, in Holmes' Surgery, American Edition.*

